# From Chaos to Care: Personalized AI for Early Cardiac Arrhythmia Warning

**DOI:** 10.64898/2026.04.08.26350403

**Published:** 2026-04-10

**Authors:** Suvankar Halder, Christopher M. Kim, Vipul Periwal

## Abstract

Cardiac arrhythmias are abnormal heart rhythms characterized by disordered electrical dynamics that impair cardiac function and pose a major global burden of morbidity and mortality. Early and accurate prediction of arrhythmic anomalies from physiological time series is crucial for effective intervention, yet remains challenging due to the nonlinear, nonstationary, and individualized nature of cardiac dynamics. Despite significant advances in machine learning-based arrhythmia detection, most existing methods operate as static classifiers on electrocardiographic signals and lack online prediction, patient-specific adaptation, and mechanistic interpretability.

From a dynamical-systems perspective, arrhythmias represent qualitative regime transitions, often preceded by subtle, temporally extended deviations that are difficult to detect in real time. Here we introduce CASCADE (Chaotic Attractor Sensitivity for Cardiac Anomaly Detection), an online and personalized anomaly forecasting framework built on a special type of reservoir computing called Dynamical Systems Machine Learning (DynML). DynML employs ensembles of continuous-time nonlinear dynamical systems as chaotic reservoirs to reconstruct and forecast short-term cardiac dynamics on a beat-to-beat basis, training only a linear readout. This design enables efficient online adaptation without retraining the underlying dynamical model. Rather than relying on static beat-level classification, CASCADE identifies arrhythmic events as failures of short-term predictability, manifested as statistically significant deviations between predicted and observed dynamics relative to subject-specific baselines.

Detection performance is governed by the intrinsic dynamical complexity of the reservoir, quantified by topological entropy. Reservoirs operating near critical entropy regimes optimally amplify subtle, temporally extended irregularities in heartbeat dynamics, rendering incipient arrhythmic signatures linearly separable at the readout level. Topological entropy thus serves both as a predictor of model performance and a principled control parameter for reservoir design. When evaluated on the MIT-BIH Arrhythmia dataset, CASCADE achieved consistently high F1 scores, precision, recall, and overall accuracy across diverse patient populations, demonstrating strong generalizability across clinical and real-world settings. By integrating chaotic reservoir computing, entropy-guided tuning, and online personalized forecasting, CASCADE reframes arrhythmia detection as a problem of dynamical regime transition rather than static classification. This perspective provides a scalable, interpretable, and computationally efficient framework for real-time cardiac monitoring and early-warning clinical decision support.

## Full Text Availability

The license terms selected by the author(s) for this preprint version do not permit archiving in PMC. The full text is available from the preprint server.

